# Impact of medical-nursing combined policy pilot on hospitalization frequency of middle-aged and older patients with chronic diseases: a quasi-experimental study based on China Health and Retirement Longitudinal Study

**DOI:** 10.3389/fpubh.2024.1450828

**Published:** 2024-10-11

**Authors:** Penghao Fan, Hongying Li, Hongyan Xu, Chao Rong

**Affiliations:** School of Humanities and Management, Zhejiang Chinese Medical University, Hangzhou, China

**Keywords:** medical-nursing combined, chronic disease, health policy, hospitalization, middle-aged and older adults

## Abstract

**Background:**

To address the growing burden of older adult care, the Chinese government has introduced a policy that integrates medical care with elder care, launching two batches of national pilot projects. A majority of the older adult population suffers from one or more chronic diseases, with many experiencing multiple chronic conditions, necessitating support from both elder care and medical services.

**Methods:**

Using panel data from the China Health and Retirement Longitudinal Study (CHARLS) spanning 2011 to 2020, this study employs the difference-in-difference (DID) model to analyze the impact of the integrated medical-nursing policy on the physical health of older patients with chronic diseases.

**Results:**

The study found that the average annual number of hospitalizations for older individuals with one or more chronic diseases was 0.276. The integrated medical-nursing policy reduced hospitalizations by 0.0405. Additionally, the average annual hospitalization rate for older individuals with two or more chronic diseases was 0.339, higher than the former group. The integrated medical-nursing policy reduced hospitalizations by 0.0738 in this group.

**Conclusion:**

The pilot study demonstrates that the implementation of the integrated medical-nursing policy has significantly improved the physical health of older patients with chronic diseases. The government should promote these policies on a larger scale, explore various forms of integrated medical care, and provide more comprehensive medical and elder care services for older patients with chronic diseases.

## 1 Introduction

As a developing country, China has to face the aging issues in most developed countries, and even become one of the countries with the fastest aging population in the world (1, 2). In 2019, there were 164.5 million Chinese citizens aged 65 and over, representing a proportion of the total population far exceeding 7% of the aging standard, meaning that China has entered an aging society (3). As the population ages, the prevalence of various chronic diseases is increasing, posing significant challenges to China’s medical system (4). Furthermore, the medical expenses of the older adults have imposed a heavy economic burden on the older adults themselves, their families, and society as a whole (5). Therefore, improving older adult care to meet the needs of an aging population requires a strong focus on the prevention and control of chronic non-communicable diseases (6).

Among the older adults with chronic diseases, many suffer from multiple chronic conditions, resulting in poorer physical and mental health (7). Focusing on this group and providing them with appropriate elder care and medical services is crucial for improving the overall health of the older adults (8). Many scholars have proposed possible solutions for chronic disease control in these populations. Studies by Shreya Kangovi, Nandita Mitra, and colleagues demonstrated that standardized community health worker interventions improved chronic disease control, mental health, quality of care, and hospitalization rates, suggesting their utility as a valuable population health management tool (9). Maud Wieczorek and Clément Meier argue that improved health literacy could empower older individuals to better manage their health, ultimately reducing the burden of chronic diseases in this population (10). Integrating primary medical resources into institutions, communities, and other elder care service systems can effectively improve the health status of older patients with chronic diseases, particularly those with multiple chronic conditions.

To control the occurrence and progression of chronic diseases, reduce the medical and pension burden on society, improve the physical and mental health of the older adults, and transform the burden of older adult care into a resource for pensions, it is essential to implement a national strategy for healthy aging. Healthy aging refers to the process by which the older adults can gradually improve their health status, reduce the incidence or slow the progression of chronic diseases, beginning with modifications to their daily living habits, thereby achieving higher benefits at a lower cost (11). Establishing a pension service system that integrates healthcare services and can function in a long-term, stable, and effective manner is a crucial measure to achieve healthy aging (12, 13).

The integration of medical care and nursing involves combining professional medical examinations with daily learning, dietary management, life care, and other essential services. Within this framework, ‘medical care’ primarily focuses on the early identification of major diseases, necessary examinations and treatments, and other medical technology services. ‘Nursing’ encompasses physical and psychological care, dietary care, daily activities, and other related services. Research by Qiu Xiaolong and Xu Xinglong indicates that the overall quality of China’s integrated medical-nursing policy is good, but it requires further optimization and improvement in terms of policy timeliness, tools, operability, and innovation (14). Zhang and Peng suggest that family pharmaceutical care can serve as a means of integrating medical care. Pharmacists utilize standardized service models to help patients resolve medication-related issues, reduce hospitalizations and medical expenses (15). With strong state support, the integrated medical-nursing model also leverages China’s rapidly developing Internet technology to enhance its operational efficiency (16, 17). The aforementioned research demonstrates that China’s integrated medical-nursing model has been widely promoted and significantly developed.

**Figure 1:**
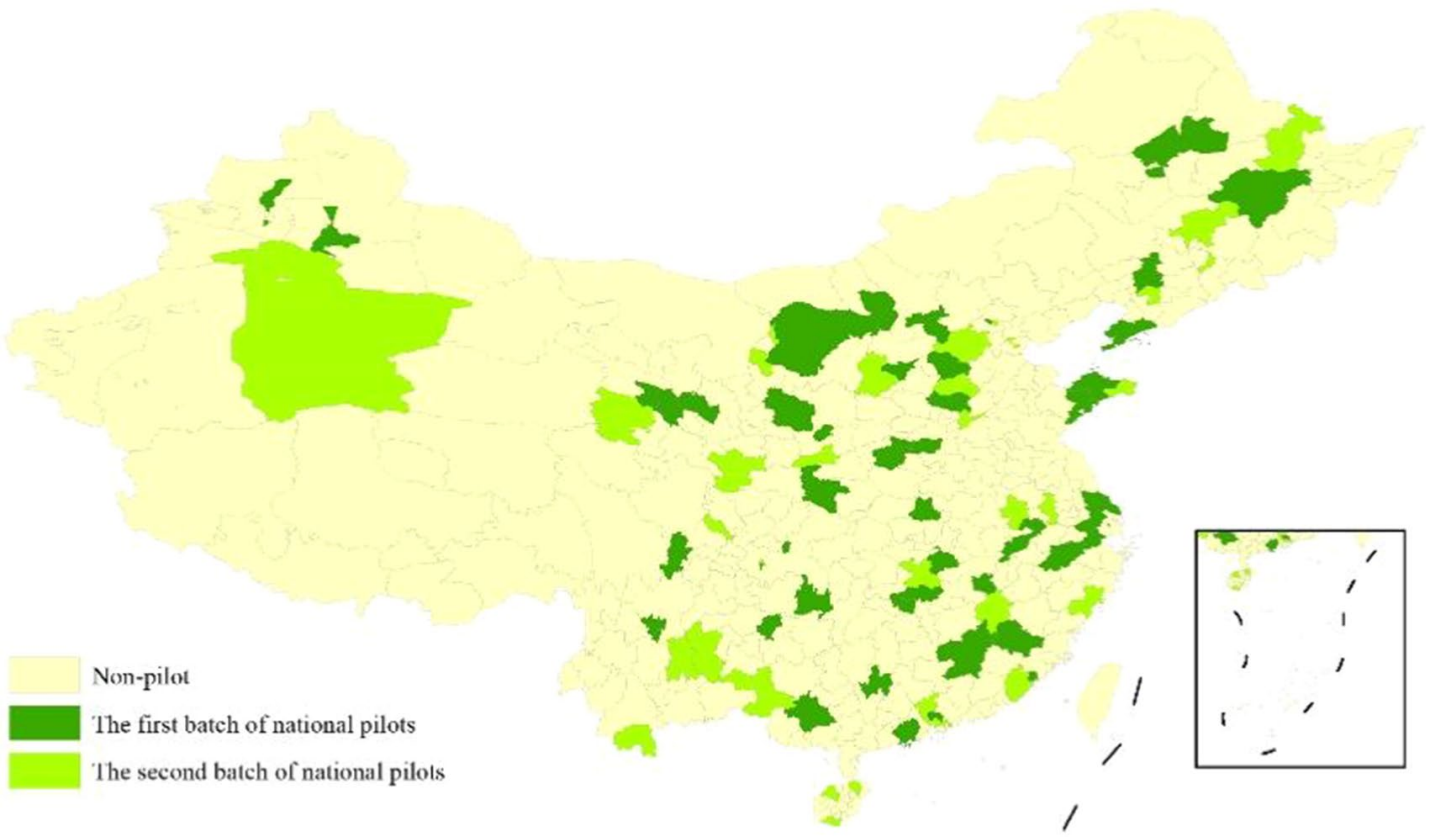
Pilot cities for the combination of medical and nursing care in China.

In 2014, the State Council issued several opinions on accelerating the development of the pension service industry, advocating for the active promotion of the integration of medical and health care with pension services. In November 2015, nine ministries and commissions of the State Council issued guidance on promoting the integration of medical and health care with pension services, clarifying key tasks. In June 2016, the National Health and Family Planning Commission and the Ministry of Civil Affairs issued the “Notice on the Determination of the First Batch of National Medical and Nursing Pilot Units,” followed by the “Notice on the Determination of the Second Batch of National Medical and Nursing Pilot Units” in September. The distribution of these two batches of pilot units is illustrated in Figure 1. The pilot units should promptly establish relevant mechanisms, fully implement the key tasks of the medical and nursing integration, and ensure that these pilots make positive progress and achieve favorable social outcomes. By December 2021, there were over 6,000 integrated medical-nursing institutions and more than 1.6 million beds nationwide. The quality of integrated medical-nursing services has significantly improved, and the older adults health support system has been gradually enhanced.

This study has two main contributions. First, China has identified two batches of national-level pilot units for the integration of medical and nursing care. Since then, the development of medical-nursing integration has been state-guided, with each region promoting its development based on local conditions. While the existing literature has evaluated the policy effect of medical and nursing integration by studying the health status of older individuals, this study subdivides the common target group of older patients with chronic diseases. We examine older patients with multiple chronic diseases as separate groups to explore the similarities and differences in policy impact compared to older patients with general chronic diseases. While existing literature has evaluated the policy effects of medical and nursing integration, this study specifically focuses on older individuals with chronic diseases. Additionally, this study examines older patients with multiple chronic diseases as separate groups to explore the similarities and differences in policy impact compared to older patients with general chronic diseases. Second, general research on the health status of the older adults predominantly uses cross-sectional data and basic regression methods. This study addresses the interference factors within the model by employing a quasi-natural experiment with the national medical-nursing integration pilot, using the DID model and the PSM-DID model to evaluate policy effects. This approach allows for a more accurate assessment of the causal relationship between the medical-nursing integration policy pilot and the health status of older patients with chronic diseases.

## 2 Materials and methods

### 2.1 Data

The data utilized in this study are sourced from the China Health and Retirement Longitudinal Study (CHARLS), a large interdisciplinary survey project conducted by the National Development Institute of Peking University and implemented by the China Social Survey Center. The objective of CHARLS is to provide a scientific foundation for effectively addressing the challenges of aging and improving policies related to the older adult population by collecting high-quality data on individuals and families among China’s middle-aged and older adult populations. CHARLS spans 150 counties and 450 communities (villages) across 28 provinces, autonomous regions, and municipalities directly under the central government. The sample comprises 19,000 respondents from a total of 12,400 households. This study uses complete data from the 2011–2020 CHARLS, encompassing the two batches of national healthcare integration pilots initiated in 2016. SPSS 26.0 and Stata 18 were used to clean the data and obtain balanced panel data for analysis. A total of 33,755 older adult samples with one or more chronic diseases were included, comprising 6,855 in the treatment group and 26,900 in the control group. Among these, 18,130 older adult samples had two or more chronic diseases, with 3,690 in the treatment group and 14,440 in the control group.

### 2.2 The explanatory variable

The explanatory variable in this study is the policy implementation of the national medical-nursing pilot units. Using the records of provinces and cities in the CHARLS data, if an individual’s city is designated as a national medical-nursing pilot, it indicates that the individual is covered by the integrated system. The CHARLS data only provides city-level records, and the national medical-nursing pilot projects in municipalities directly under the central government are implemented at the district level. We regard the four municipalities directly under the central government as pilot areas for medical-nursing integration, after considering the resource richness and accessibility across districts within these municipalities. The five-period data from CHARLS are collected at specific intervals. Therefore, this study analyzes data from these specific periods to assess the effects of the policies.

### 2.3 The explained variable

The explained variable in this study is the frequency of hospitalization, specifically the number of hospitalizations within 1 year. The question “How many times have you received inpatient care during the past year?” enables us to determine the number of hospitalizations an individual had within 1 year. The number of hospitalizations effectively reflects the health status and chronic disease management of older patients with chronic conditions and comorbidities. It is a sensitive indicator that can demonstrate the impact of policies on the health status of the older adults (18).

### 2.4 The control variables

To exclude the impact of other variables on the study, we selected basic demographic information and other potentially influential variables as control variables, including sex, age, marital status, education level, pension insurance, medical insurance, and social activities. In this study, education level is categorized into four grades: ‘illiterate or unfinished primary school’, ‘primary school’, ‘secondary school’, and ‘college or undergraduate and above’. The social activities in the questionnaire encompassed 11 basic social activities, including interaction with friends. The definition of other control variables are presented in Table 1.

**Table 1:** Descriptive statistics.

(A) Older adults people with general chronic diseases							
Variables	Variable definition	All samples		Treatment group		Control group	
		Mean	SD	Mean	SD	Mean	SD
Inpatient frequency		0.276	0.784	0.270	0.718	0.278	0.800
Sex	Male = 1, Female = 0	0.443	0.497	0.434	0.496	0.445	0.497
Age	Interview year minus birth year	62.746	9.286	62.887	9.102	62.710	9.332
Marital status	Spouse = 1, No spouse = 0	0.855	0.352	0.864	0.342	0.853	0.354
Educational level	Record of formal schooling	1.823	0.889	1.968	0.910	1.787	0.880
Endowment insurance	Yes = 1, No = 0	0.683	0.465	0.692	0.462	0.681	0.466
Medical insurance	Yes = 1, No = 0	0.955	0.207	0.960	0.197	0.954	0.209
Social activity	Participation in one or more of the all activities = 1, Participation in none = 0	0.521	0.500	0.541	0.498	0.516	0.500

(B) Older adults people with multiple chronic diseases							
Variables	Variable definition	All samples		Treatment group		Control group	
		Mean	SD	Mean	SD	Mean	SD
Inpatient frequency		0.339	0.843	0.318	0.776	0.344	0.860
Sex	Male = 1, Female = 0	0.424	0.494	0.424	0.494	0.424	0.494
Age	Interview year minus birth year	63.371	9.229	63.812	8.920	63.258	9.303
Marital status	Spouse = 1, No spouse = 0	0.846	0.361	0.854	0.353	0.844	0.363
Educational level	Record of formal schooling	1.807	0.891	1.962	0.918	1.768	0.879
Endowment insurance	Yes = 1, No = 0	0.685	0.465	0.686	0.464	0.684	0.465
Medical insurance	Yes = 1, No = 0	0.958	0.200	0.961	0.194	0.957	0.202
Social activity	Participation in one or more of the all activities = 1, Participation in none = 0	0.524	0.499	0.557	0.497	0.516	0.500

### 2.5 DID model

The distribution of national medical-nursing pilot units across most provinces provides a quasi-natural experiment opportunity for this study. In this study, the difference-in-difference (DID) model was used to evaluate the impact of the integrated medical-nursing policy on the health status of older individuals with chronic diseases. Unlike traditional regression models, the DID model has strong applicability and effectively avoids endogeneity problems caused by missing variables and selection bias. In addition, the traditional regression model cannot generate accurate causal inference. Therefore, this commonly used method does not have the ability to accurately assess the effectiveness of policies. As an important method of group causal effect estimation, DID has been used by more and more scholars as the preferred method to evaluate the effect of policy because of its multiple and complete test process. The number and distribution characteristics of the national medical-nursing pilot units are also important determinants of the DID model used in this study. In accordance with the requirements of the DID model, this study constructs two sets of dummy variables. The first set is the policy dummy variable (Treat), including the ‘treatment group’ affected by the policy and the ‘control group’ not affected by the policy. If the city where the older adults with chronic diseases reside is included in the national medical-nursing pilot, the value is assigned 1; otherwise, it is assigned 0. The second set is the time dummy variable (Time), representing the periods before and after the policy implementation. Before the policy implementation, the value is 0, and after the policy implementation, the value is 1. By comparing the differences of relevant indicators between the treatment and control groups before and after policy implementation, the improvement in health status of the older adults with chronic diseases due to the integrated medical-nursing policy was systematically analyzed. Therefore, this study constructs a DID model:


$$IN_{ict}=\beta_{0}+\beta_{1}\mathrm{Trea}t_{ic}\times Time_{t}+\beta 2\sum Z_{\mathrm{it}}+\mu_{i}+\tau_{t}+\varepsilon_{ict}$$

Among them, $IN_{ict}$ represents the mental health status of individual i living in city c during period t, and the variable is the number of hospitalizations within 1 year. Among them, $\mathrm{Trea}t_{ic}\times Time_{t}$ is the core explanatory variable, and $\beta_{1}$ represents the policy effect of the integrated medical-nursing care. $\sum Z_{\mathrm{it}}$ represents a series of control variables, β2 represents the corresponding regression coefficient, $\beta_{0}$ is the intercept term, $\mu_{i}$ represents the individual fixed effect, $\tau_{t}$ represents the time fixed effect, and $\varepsilon_{ict}$ represents the random disturbance term.

In addition, we use the PSM-DID method to test the robustness. As a quasi-natural experiment, policy implementation in reality inevitably introduces selection bias in the DID model. The PSM method can approximate randomization in the quasi-natural experiment. Therefore, after controlling the covariates, we matched the treatment and control groups based on similar or identical scores to eliminate selection bias, thereby more accurately evaluating the impact of the integrated medical-nursing care on the health status of older individuals with chronic diseases. On this basis, this study constructs the PSM-DID model as follows:


$$IN_{ict}^{PSM}=\beta_{0}+\beta_{1}\mathrm{Trea}t_{ic}\times Time_{t}+\beta 2\sum Z_{\mathrm{it}}+\mu_{i}+\tau_{t}+\varepsilon_{ict}$$

## 3 Results

### 3.1 Descriptive statistical analysis

The results of the descriptive statistical analysis in this study are presented in Table 1. The average annual number of hospitalizations for all samples of older patients with chronic diseases was 0.276. For older patients with multiple chronic diseases, the average annual number of hospitalizations was higher at 0.339. Notably, in both groups, the hospitalization frequency in the treatment group was lower than that in the control group. The decline in hospitalization frequency among older individuals with chronic diseases is more pronounced. This suggests that older individuals in the pilot areas of the integrated medical-nursing policy may have better physical health. However, further verification and testing are required. Regarding other control variables, the average age of the older adults with one or more chronic diseases is 62.746, which is slightly lower than the average age of those with two or more chronic diseases, at 63.371. In terms of sex distribution, both groups have a higher proportion of females. Most samples in both groups are married; the average education level is below primary school, but close to primary school. The medical insurance coverage rates in the two groups was 95.5 and 95.8%, respectively. The pension insurance coverage rates are lower than those of medical insurance, at 68.3 and 68.5%, respectively. The proportion of older individuals participating in social activities is relatively close to those not participating in social activities. Additionally, comparison between groups revealed that the mean values for age, marital status, education level, pension insurance, medical insurance, and social activities were higher in the treatment group than in the control group.

### 3.2 Main regression results

The impact of the national medical-nursing combined pilot on the hospitalization frequency of the older adults is shown in Table 2. Table 2 presents the crude regression results for the two groups after accounting for individual fixed effects and time fixed effects, as well as the regression results after controlling for other variables. The results indicate that the policy implementation in the pilot areas significantly reduced the frequency of hospitalizations among older patients with chronic diseases. Moreover, the reduction in hospitalization frequency is more pronounced among older patients with two or more chronic diseases. Specifically, policy implementation in the pilot areas reduced the number of annual hospitalizations by 0.0405 times for the first group and 0.0738 times for the second group. Additionally, among the control variables, increasing age and being unmarried were associated with higher annual hospitalization rates for older patients with chronic diseases. No significant correlation was found between other control variables and hospitalization frequency.

**Table 2:** Main regression results.

(A) Older adults people with general chronic diseases						
Variables	Crude			Adjusted		
	Coeff	Std. err.	*t*	Coeff	Std. err.	*t*
Treat × Time	−0.0407	0.019	−2.10^**^	−0.0405	0.019	−2.08^**^
Sex				−0.0647	0.091	−0.71
Age				0.0062	0.003	1.99^**^
Marital status				−0.0726	0.026	−2.82^***^
Educational level				0.0096	0.016	0.59
Endowment insurance				0.0050	0.012	0.40
Medical insurance				0.0199	0.022	0.90
Social activity				−0.0054	0.010	−0.54
_cons	0.1433	0.009	16.73^***^	−0.1604	0.193	−0.83
Time-fixed effect	Yes			Yes		
Individual fixed effect	Yes			Yes		

(B) Older adults people with multiple chronic diseases						
Variables	Crude			Adjusted		
	Coeff	Std. Err.	*t*	Coeff	Std. Err.	*t*
Treat × Time	−0.0739	0.028	−2.60^***^	−0.0738	0.028	−2.59^***^
Sex				−0.0178	0.135	−0.13
Age				0.0089	0.004	2.01^**^
Marital status				−0.0815	0.037	−2.23^**^
Educational level				−0.0207	0.024	−0.85
Endowment insurance				−0.0152	0.018	−0.84
Medical insurance				−0.0164	0.033	−0.49
Social activity				−0.0086	0.015	−0.58
_cons	0.1921	0.013	15.34^***^	−0.1919	0.275	−0.70
Time-fixed effect	Yes			Yes		
Individual fixed effect	Yes			Yes		

^*^p < 0.1, ^**^p < 0.05, ^***^p < 0.01.

### 3.3 Equilibrium trends and dynamic effects tests

**Figure 2:**
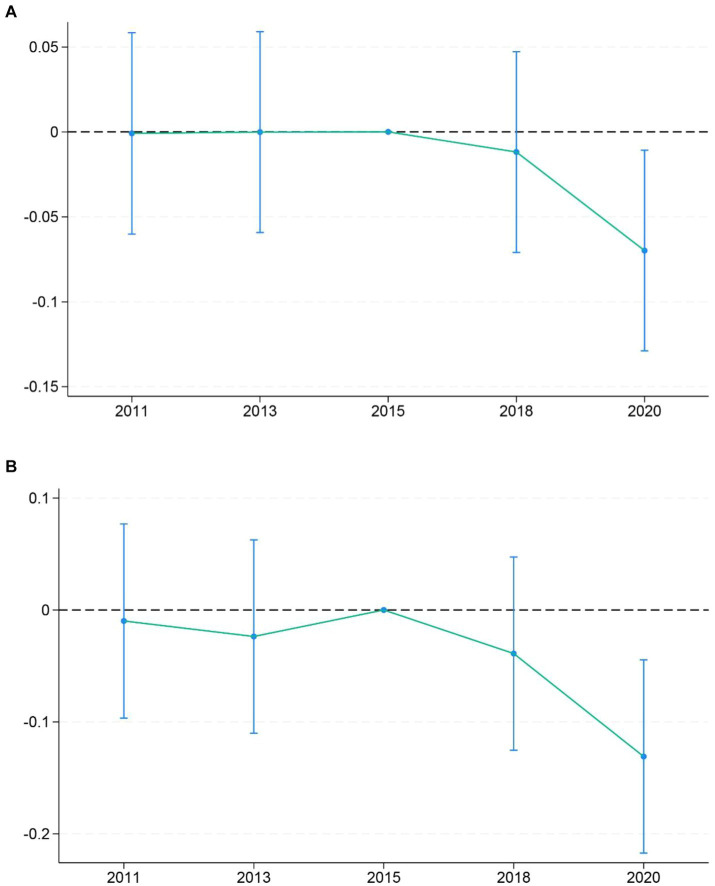
Equilibrium trends and dynamic effects tests. **(A)** Elderly people with general chronic diseases. **(B)** Elderly people with multiple chronic diseases.

A basic premise for using the DID model to evaluate policy effects is that the development trends of the explained variables for the treatment and control groups should be consistent before policy implementation in the pilot. In other words, there should be no statistically significant difference between the treatment and control groups before policy implementation. Therefore, it is necessary to conduct a parallel trend test for the DID model. In this study, we use the period prior to policy implementation, specifically 2015, as the reference group. We incorporate the interaction term of the policy dummy variable and the time dummy variable into the regression equation, and ultimately obtain the confidence interval diagram for the parallel trend and dynamic effect test as shown in Figure 2. As shown in Figure 2, there was no significant difference in the average annual number of hospitalizations between policy pilot cities and non-policy pilot cities in both groups before 2015. In 2018, the first period after policy implementation, there was no significant difference in the average annual number of hospitalizations between policy pilot cities and non-policy pilot cities, which may be attributed to policy lag. However, in the second period after policy implementation, namely in 2020, the hospitalization frequency of older individuals living in the pilot cities for national medical-nursing care significantly decreased. It indicates that the DID model in this study passes the parallel trend test, thereby validating the regression results presented in Table 2 to some extent.

### 3.4 Robustness tests

This study also conducted a series of robustness tests on the regression results shown in Table 2. First, the PSM-DID model constructed above is tested. After processing the samples using propensity score matching, DID model regression analysis was conducted to address endogeneity problems caused by changes in individual characteristics in the treatment and control groups. The influence of other factors was excluded to ensure the analysis results more accurately reflect the policy effect itself. To minimize selection bias, individuals with similar or identical scores in the control variables were matched between the treatment and control groups. The standardized differences of the variables before and after the matching are shown in Table 3. Before matching, the total sample of older individuals with one or more chronic diseases showed significant differences within the 95% confidence interval in terms of sex, age, education level, medical insurance, and social activities. Similarly, the total sample of older patients with two or more chronic diseases showed significant differences in age, education level, and social activities within the 95% confidence interval. After matching, the deviation in all control variables between the two groups was reduced to less than 10%, achieving the goal of minimizing selection bias. The DID results after PSM matching are presented in Table 4. It is evident that the integrated medical-nursing policy significantly reduces hospitalization frequency in both groups, supporting the robustness of the results in Table 2.

**Table 3:** Balance test results of PSM-DID model.

(A) Older adults people with general chronic diseases								
Variables	Before matching				After matching			
	Control group	Treatment group	*t*	%bias	Control group	Treatment group	*t*	%bias
Sex	0.469	0.451	−2.66^***^	−3.6	0.452	0.451	−0.17	−0.3
Age	61.320	61.585	1.97^**^	2.7	61.663	61.587	−0.46	−0.8
Marital status	0.858	0.858	−0.03	<−0.1	0.866	0.858	−1.32	−2.2
Educational level	1.821	2.044	17.95^***^	24.1	2.040	2.044	0.26	0.5
Endowment insurance	0.546	0.558	1.78^*^	2.4	0.558	0.558	0.07	0.1
Medical insurance	0.941	0.948	2.29^**^	3.2	0.955	0.948	−1.68^*^	−2.7
Social activity	0.555	0.597	6.23^***^	8.5	0.597	0.597	−0.03	−0.1

(B) Older adults people with multiple chronic diseases								
Variables	Before matching				After matching			
	Control group	Treatment group	*t*	%bias	Control group	Treatment group	*t*	%bias
Sex	0.424	0.425	0.06	0.1	0.418	0.425	0.43	1.3
Age	60.901	61.436	2.56^**^	6.2	61.607	61.424	−0.72	−2.1
Marital status	0.873	0.878	0.73	1.8	0.890	0.878	−1.18	−3.4
Educational level	1.776	1.973	9.25^***^	21.7	1.956	1.971	0.51	1.6
Endowment insurance	0.551	0.550	−0.09	−0.2	0.556	0.550	−0.36	−1.1
Medical insurance	0.956	0.954	−0.30	−0.7	0.970	0.954	−2.68^***^	−7.4
Social activity	0.543	0.597	4.54^***^	10.9	0.600	0.597	−0.22	−0.6

^*^p < 0.1, ^**^p < 0.05, ^***^p < 0.01.

**Table 4:** Results of the robustness tests.

(A) Older adults people with general chronic diseases						
Test method	Crude			Adjusted		
	Coeff	Std. err.	*t* (*z*)	Coeff	Std. err.	*t* (*z*)
(1) PSM-DID test						
	−0.0762	0.023	−3.30^***^	−0.0751	0.023	−3.25^***^
(2) Replacement variable method						
Number of chronic diseases	−0.0406	0.019	−2.09^**^	−0.0417	0.019	−2.14^**^
(3) Adjusting fixed effects						
Community × year fixed effect	−0.0413	0.019	−2.12^**^	−0.0411	0.019	−2.11^**^

(B) Older adults people with multiple chronic diseases						
Test method	Crude			Adjusted		
	Coeff	Std. err.	*t* (*z*)	Coeff	Std. err.	*t* (*z*)
(1) PSM-DID test						
	−0.0753	0.028	−2.65^***^	−0.0752	0.028	−2.64^***^
(2) Replacement variable method						
Number of chronic diseases	−0.1026	0.032	−3.25^***^	−0.1051	0.032	−3.32^***^
(3) Adjusting fixed effects						
Community × year fixed effect	−0.0736	0.028	−2.59^**^	−0.0751	0.028	−2.64^***^

^*^p < 0.1, ^**^p < 0.05, ^***^p < 0.01.

Second, this study employs the method of substitution variables to test robustness. The accumulation of chronic diseases can naturally lead to a decline in individual health status, potentially increasing the number of hospitalizations (19). The older adults additionally, the number of chronic diseases and the frequency of hospitalization are key indicators of the health status of the older adults. Therefore, we use the number of chronic diseases as a substitution variable for hospitalization frequency and incorporate it into the regression model. The results are shown in Table 4. Thus, it can be concluded that the national medical-nursing integrated policy pilot effectively controls the number of chronic diseases in older individuals with chronic conditions.

Third, we also adjusted the fixed effects. In the baseline regression results, individual and time fixed effects were controlled. To eliminate the influence of community differences, we adjusted the fixed effects to account for community and time fixed effects, and then performed regression analysis. The results still indicate that the integrated medical-nursing policy significantly reduces hospitalization frequency in both groups, validating the baseline regression results.

### 3.5 Placebo test

**Figure 3:**
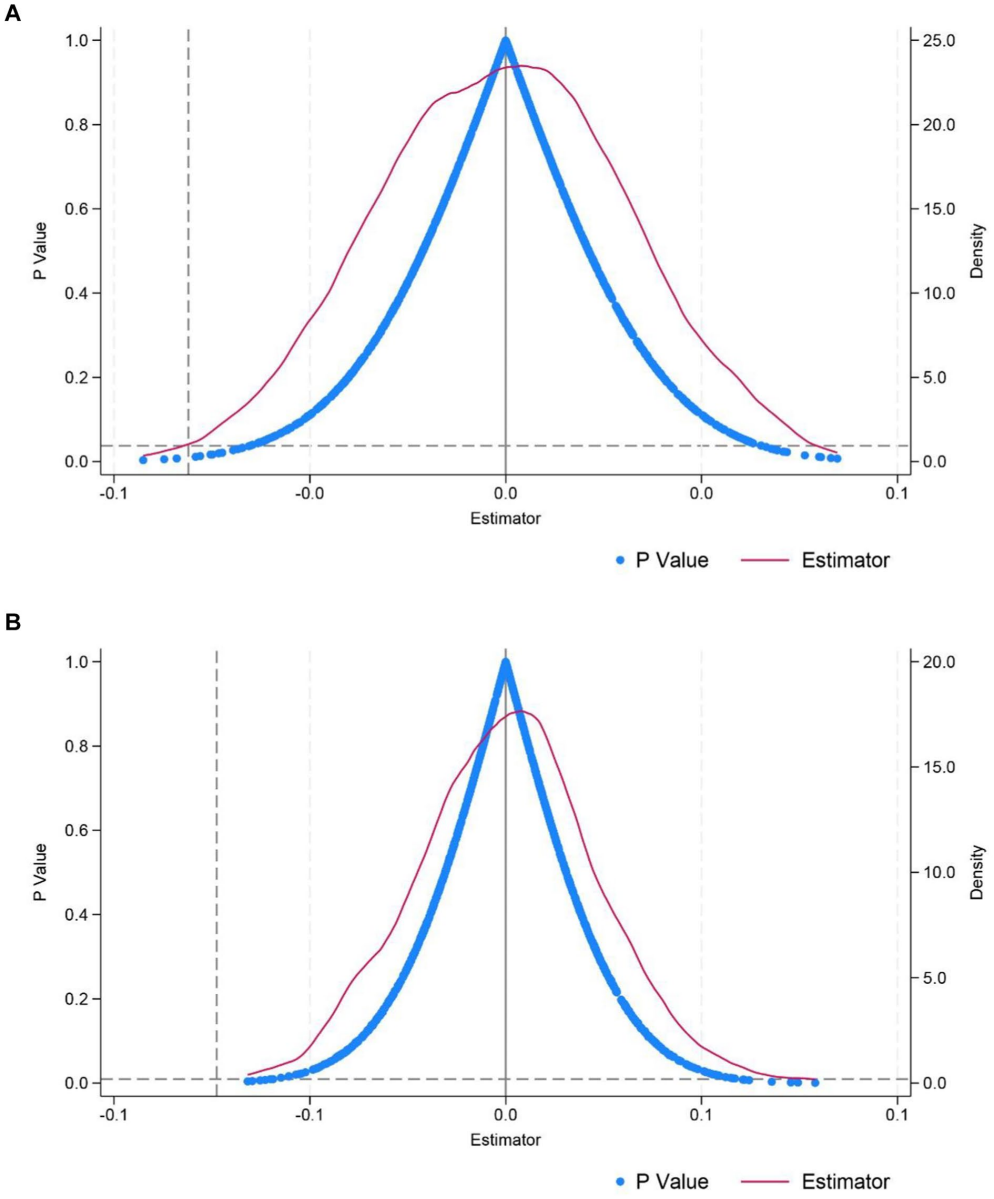
Placebo test. **(A)** Elderly people with general chronic diseases. **(B)** Elderly people with multiple chronic diseases.

The basic principle of placebo test on policy effects is similar to that of a placebo in medicine: using false policy occurrence times or experimental groups to test whether observed policy effects can be attributed to the policy itself. If a policy effect is still observed, it indicates that the policy effect in the benchmark regression is unreliable and may be caused by other unobservable factors rather than the policy itself. In this study, 1,000 random samples were conducted on the interaction between the policy dummy variable and the time dummy variable to generate a kernel density estimation map of the interaction coefficient and *p* value, as shown in Figure 3, to verify the policy effect.

Figure 3 shows that most of the coefficients are concentrated near 0 and do not exceed the true value indicated by the vertical dotted line. Simultaneously, most of the estimated coefficients are not lower than the horizontal dotted line, indicating they are not significant. This indicates that the policy effect of the national medical-nursing integrated policy pilot on the hospitalization frequency of older patients with chronic diseases is not influenced by other unobservable factors.

## 4 Discussion

This study found that the implementation of the national medical-nursing combined policy pilot significantly reduces the number of hospitalizations for older individuals with chronic diseases. Additionally, the policy pilot has a more pronounced effect on reducing the hospitalization frequency of older patients with chronic disease complications. This indicates that older individuals with multiple chronic diseases can improve their health status through the integration of medical and pension services. Risk factors for one chronic disease, such as body mass index, may also be risk factors for other chronic diseases (20). Moreover, some chronic diseases themselves are risk factors for other chronic diseases (21). Therefore, a single chronic disease can easily develop into multiple chronic diseases, and the older adults with multiple chronic diseases may lead to a rapid decline in health due to the combined symptoms of these diseases. Consequently, older individuals with chronic diseases often have complex needs that require the coordination of multiple professionals and services for effective treatment and care (22). China’s integrated medical-nursing model not only provides pension services for this group but also delivers urgently needed healthcare services that bring practical benefits. Additionally, we found that unmarried individuals have a higher frequency of hospitalization and lower health status, consistent with the findings of scholars such as David Cantarero-Prieto and Marta Pascual-Sáez (23). The relationship between age and the health of patients with chronic diseases aligns with the research of Mayra Tisminetzky and Christopher Delude et al., who suggest that a more comprehensive understanding of the impact of comorbidity burden and aging in older patients is necessary (24).

This study uses hospitalization frequency to reflect the health status of older patients with chronic diseases and to a certain extent, the medical burden on this population. YANG Jian, LI Yuan-qing, and other scholars assert that the hospitalization burden of middle-aged and older patients with chronic diseases in China is growing rapidly (25). Although China has achieved extensive medical insurance coverage, as reflected in this study, the medical expenses associated with hospitalization still pose a significant burden for many older individuals, especially those in rural areas, when compared to their lower pension insurance coverage and disposable income (26, 27). Furthermore, scholars such as Shan Gao and Shasha Sun have found that comorbidity has become a significant issue for older inpatients (28). China’s severe aging population and high prevalence of chronic diseases among the older adults indicate that without an effective response, the country will face a substantial pension and medical burden in the future (29). Controlling the progression of chronic diseases in the older adults through various primary healthcare services to prevent condition deterioration and subsequent hospitalization can effectively alleviate these burdens. Therefore, this study indicates that the decline in hospitalization frequency among older patients with chronic diseases in the pilot areas suggests that this policy can alleviate future pension and medical burdens in China to some extent. The combination of medical and nursing services can integrate primary medical services into the daily life of the older adults. Professional health care personnel cultivate healthy living habits for older patients with chronic diseases through professional knowledge training, so as to realize the advancement of health services. At the same time, this prevention-oriented health service model can significantly reduce the severity of chronic diseases in older individuals and the incidence of chronic diseases in groups. This can have a positive mitigation effect on the family burden of the older adults and the overall social burden. The large-scale promotion of this policy could provide more support to older individuals and bring greater benefits to society as a whole.

In recent years, the degree of population aging in China has been increasing. Various chronic diseases have emerged among the older adults, with multiple chronic conditions becoming more common. Therefore, enhancing medical services tailored to the older adults can improve their health status. Moreover, providing daily medical services can prevent the malignant progression of chronic diseases in the older adults, reduce the incidence of other chronic conditions, decrease the frequency of medical treatments and associated expenses, thereby reducing the personal burden on the older adults and the overall pension burden on society. Thus, the integration of medical care and nursing is particularly important in China’s pension industry. The integrated medical-nursing model can combine the strengths of each service, compensate for the shortcomings of implementing a single service, and provide appropriate and comprehensive care for older individuals with chronic diseases (30). Separate old-age care services lack professional doctors and nurses to provide targeted medical, rehabilitation, and healthcare services for individuals with chronic diseases, who constitute the majority of the older adults. They cannot effectively control disease progression by adjusting daily living habits such as diet and routine. Separate medical services for older patients with chronic diseases focus solely on medical aspects, often neglecting changes in individual needs and mental health status. This oversight can lead to issues such as depressive symptoms or cognitive decline, deviating from the goal of healthy aging. Additionally, it is impossible to achieve the goal of transforming the burden of older adult care into a resource at the national level. Combining medical and nursing care can yield substantial social benefits, and the comprehensive promotion of this policy may benefit older individuals nationwide.

It is worth noting that some scholars have put forward different views on the policy of combining medical and nursing care. Mariona and others believe that the development of integrated medical and nursing services often requires the advice and support of experts in medical and care services. This is more difficult for rural areas (31). Tao and other scholars believe that the older adults in the medical and nursing institutions do not have good health literacy through their investigation and research, and need to strengthen health education for the older adults (32). At the same time, there is a lack of effective measurement tools to assess the satisfaction of the older adults with integrated medical and nursing services (33). In addition, through empirical analysis, some scholars believe that there are problems such as lack of legal policies, fragmentation of management, and mismatch between supply and demand of services in the practice of medical and nursing integration (34, 35). These studies show that China’s medical-nursing combined policy is still in the exploratory stage. Although the implementation of the policy to a certain effect, there are still many areas for improvement.

## 5 Conclusion and recommendations

The results of this study demonstrate that the national medical-nursing combined policy pilot significantly reduces hospitalization frequency among older patients with chronic diseases and improves their health status. The positive effects of the policy indicate the need for further refinement and promotion. This policy is not only crucial for reducing the social pension burden but also for achieving the goal of “prevention first” in public health to a significant extent. For older individuals with slight abnormal indicators but no symptoms of chronic diseases, adjusting their living habits can prevent the onset of chronic conditions. For older individuals who have developed chronic diseases, especially those with multiple conditions, disease progression can be controlled through diet and medication, thereby avoiding worsening disease and frequent hospitalizations.

Currently, some scholars have noted that the integration of medical care and nursing in China has developed rapidly, with four typical models emerging: ‘home medical care’, ‘community medical care’, ‘institutional medical care’ and ‘comprehensive medical care’ (35). The emergence of these models demonstrates the government’s strong support for and promotion of the integrated medical-nursing policy. However, each model faces certain developmental challenges. According to Shangren Qin, Mengqiu Zhou, and colleagues, the most preferred model of integrated medical and nursing care for the older adults in China is home-based medical care (36). This preference may be due to the combination of thrifty living habits and traditional values formed by the older adults through their life experiences. The effectiveness of this home-based medical care model primarily depends on the signing of family doctors, but the issue of “signing without service” frequently arises. Therefore, it is necessary not only to popularize contracted family doctor services but also to implement supervision over the performance and responsibilities of family doctors. Additionally, the ‘institutional medical care’ model is strongly supported by local government civil affairs departments, with some regions developing models like ‘healthcare combination’ based on this approach. Research by some scholars has found that older individuals choosing institutions for integrated medical and nursing services prioritize service quality and medical technology levels. They are more willing to spend money on these aspects (37), providing insights for the future development of integrated medical and nursing institutions. Compared to improving service quality, enhancing medical technology levels is much more challenging. Improving salaries and treatment to recruit more skilled doctors and collaborating with medical institutions to open a ‘green channel’ is an effective measure. Additionally, the government should take the lead in promoting cooperation, providing policy support, and offering tax relief to reduce the operational pressure on institutions when development challenges arise.

## 6 Limitations

There are some limitations in this study. Firstly, the national medical-nursing combined policy pilot implementation in municipalities directly under the central government is conducted at the district and county levels, but we can only obtain urban-level residence information from the CHARLS database. Therefore, considering the resource richness of municipalities directly under the central government and the ease of transportation between districts and counties, we use the entire municipality as a national pilot for the integration of medical care and nursing. Secondly, our initial aim was to explore the impact of the medical-nursing combined policy on the annual hospitalization expenses of older patients with chronic diseases. It is regrettable that the questionnaire for the fifth wave of CHARLS data in 2020 has been deleted compared with the previous waves. Some of the details of the hospitalization are not available in the 2020 data. Contents related to hospitalization information for middle-aged and older adults, such as hospitalization expenses, hospitalization days, and reasons for hospitalization, can only be tracked until 2018. After comprehensive consideration, we finally chose the number of annual hospitalizations of individuals as the dependent variable of this study. If there is an opportunity, we hope to conduct more comprehensive and in-depth research through field research in the future.

## Data Availability

Publicly available datasets were analyzed in this study. This data can be found here: https://charls.pku.edu.cn/gy/gyxm.htm.
